# Reflections on a person's experience of mental illness: an innovative teaching pilot for second-year medical students

**DOI:** 10.1192/bjo.2021.82

**Published:** 2021-06-18

**Authors:** George Blanchard, Louis Quail, Grace Yang, Katherine Terence, Amisha Kalra, Neil Sarkar, Aimee Spector, Seri Abraham, Suzanne Reeves

**Affiliations:** 1 UCL Medical School, Camden and Islington NHS Foundation Trust; 2 UCL Medical School; 3 Camden and Islington NHS Foundation Trust; 4 University College London; 5 Manchester Metropolitan University, Health Education North West, Royal Oldham Hospital, Oldham, Pennine Care NHS Foundation Trust; 6 University College London, Camden and Islington NHS Foundation Trust

## Abstract

**Aims:**

We sought to develop a teaching pilot to help year 2 medical students meet the following learning outcomes: Develop a better understanding of patient and carer experiences of mental illness; Recognise and challenge unhelpful attitudes towards people with mental illness; Promote a broader understanding of cultural issues surrounding mental illness, including stigma and discrimination.

**Method:**

337 medical students were invited to attend a lecture by author LQ, a documentary photographer who presented a narrative of his brother Justin's lived experience of schizophrenia (louisquail.com/big-brother-introduction). 197 students attended the session, which was recorded and made available online. Students were invited to enter a competition to win a signed copy of LQ's book, ‘Big Brother’ and asked to submit either a 500-word written reflective piece, or a creative work accompanied by a 200-word statement. 13 submissions were received, including paintings, drawings, collage, photography, and poetry, all of which were blind rated by authors SR and GB, based on originality and quality of reflection. Of the six shortlisted, three winning entries were chosen by author LQ.

**Result:**

All reflections moved away from a technical understanding of schizophrenia, towards person-centred interpretations, with dominant themes of ‘stigma’, ‘disempowerment’, ‘understanding people as individuals’, ‘subjective experience of mental illness’, ‘inclusion’ and ‘healing power of nature’.

The three prize winners (authors GY, AK and KT) used different mediums: GY painted an osprey over a chaotic collage of disordered and stigmatizing words (the osprey representing empowerment and the “reservoir for wellbeing in nature”); AK's sonnet began as an ode to the chaos of Justin's experience, but the concluding lines reframed this struggle, conveying feelings of hope and beauty; and KT's self-portrait, produced with a slow shutter-speed photograph, powerfully conveyed a sense of disorientation and disturbance. She reflected on how the stigma of mental illness affects self-perception. The talk was well-attended, and reflections were of high quality. A limitation of this pilot was that only a small proportion of students completed the reflective assignment.

**Conclusion:**

Innovative teaching strategies are needed to address negative attitudes towards mental illness and psychiatry, which are prevalent amongst the medical profession. This pilot provides a model for combining carer-led, reflective, and creative elements in undergraduate psychiatry teaching, with the aim of challenging stigma. This model will be evaluated in a further study involving fifth year medical students, which will use a validated scale to measure change in students’ attitudes towards mental illness and psychiatry.

